# Gonadotropin-Releasing Hormone Receptor-Targeted Near-Infrared Fluorescence Probe for Specific Recognition and Localization of Peritoneal Metastases of Ovarian Cancer

**DOI:** 10.3389/fonc.2020.00266

**Published:** 2020-02-28

**Authors:** Qiyu Liu, Xiaobo Zhou, Wei Feng, Tao Pu, Xiaoping Li, Fuyou Li, Yu Kang, Xiaoyan Zhang, Congjian Xu

**Affiliations:** ^1^Obstetrics and Gynecology Hospital, Fudan University, Shanghai, China; ^2^Department of Obstetrics and Gynecology of Shanghai Medical School, Fudan University, Shanghai, China; ^3^Shanghai Key Laboratory of Female Reproductive Endocrine Related Diseases, Shanghai, China; ^4^Department of Chemistry, Fudan University, Shanghai, China; ^5^Department of Obstetrics and Gynecology, Peking University People's Hospital, Beijing, China

**Keywords:** targeted imaging, near-infrared fluorescence, gonadotropin-releasing hormone receptor, indocyanine green, ovarian cancer

## Abstract

**Background:** Peritoneal dissemination is common in advanced ovarian cancer. The completeness of cytoreduction is an independent prognostic factor. The intraoperative fluorescence imaging via tumor-specific near-infrared fluorophore might improve staging and surgical completeness. A promising target for ovarian cancer is the gonadotropin-releasing hormone receptor (GnRHR). This study aimed to develop a GnRHR-targeted near-infrared imaging probe for the detection of peritoneal metastases of ovarian cancer.

**Methods:** Indocyanine green (ICG) was conjugated with GnRH antagonist peptide to develop an ovarian cancer-selective fluorescence probe GnRHa-ICG. GnRHR expression was detected in ovarian cancer tissues. The binding capacity of GnRHa-ICG and ICG was detected in both cancer cell lines and mouse models of peritoneal metastatic ovarian cancer using fluorescence microscopy, flow cytometry, and near-infrared fluorescence imaging.

**Results:** Tissue microarray analysis revealed the overexpression of GnRHR in ovarian cancer. GnRH-ICG exhibited the binding capacity in a panel of cancer cell lines with different expression levels of GnRHR. In ovarian cancer mouse models, GnRHa-ICG signals were detected in peritoneal tumor lesions rather than normal peritoneal and intestines tissues. ICG showed intensive fluorescence signals in intestines. The tumor-to-muscle ratio and tumor-to-intestine ratio of GnRHa-ICG was 7.41 ± 2.82 and 4.37 ± 1.66, higher than that of ICG (4.60 ± 0.50 and 0.57 ± 0.06) at 2 h post administration. The fluorescence signal of peritoneal metastases peaked in intensity at 2 h and maintained for up to 48 h. ICG also showed a weak signal in the tumor lesions due to the enhanced permeability and retention effect, but the intensity decreased quickly within 48 h.

**Conclusions:** The developed GnRHR-targeted imaging agent GnRHa-ICG could specifically detected peritoneal tumor lesions from normal peritoneal and intestines tissues because of the modification of GnRHa to ICG. The plateau period of GnRHa-ICG accumulation may be feasible for clinical applications in fluorescence-guided surgery. Our GnRHR imaging concept may be effective in other hormone-related tumors with upregulated GnRHR expression.

## Introduction

Among all gynecologic malignancies, ovarian cancer is the most common cause of death worldwide (1). The 10-year survival rate is only 15% for patients diagnosed with stage III-IV disease (2). Unfortunately, over 80% of cases are diagnosed at advanced stages. Currently, cytoreductive surgery remains the cornerstone of treatment for advanced ovarian cancer. The completeness of cytoreduction is associated with local recurrence rates and clinical outcomes (3). The median survival time of patients who undergo complete resection of all visible disease (R0) is 99.1 months, and the corresponding durations for patients with residuals of 0.1–1 cm or >1 cm are 36.2 or 29.6 months, respectively (4). Patients with R0 resection seem to have the best overall outcomes (5). However, the R0 resection rate is only 8.1% in patients with stage IV epithelial ovarian cancer and 51.2% in patients with stage IIIC or IV ovarian cancer after neoadjuvant chemotherapy (6, 7). Successful cytoreduction relies on the accurate localization of cancerous lesions, especially minimal residual diseases, followed by their complete resection.

Traditional imaging approaches, such as ultrasound, CT, and MRI, are more suitable for pre- and post-operative assessment. The combination of real-time fluorescence imaging and tumor-targeted fluorophores can transform the paradigm of surgery through the accurate intraoperative differentiation of cancer from adjacent normal tissue (8). Such an approach might improve staging and survival rates.

Over the past two decades, researchers have attempted to improve the fluorescence imaging system, identify potential targetable biomarkers, and develop tumor-specific fluorophores for clinical applications (9–11). In particular, near-infrared fluorescence (NIRF) imaging is advantageous for clinical use owing to its improved penetration depth and limited autofluorescence compared with visible-light fluorescence imaging (12). Indocyanine green (ICG) is the first FDA-approved near-infrared fluorophore and has been widely used in clinical angiography and lymphography. Through binding with serum proteins, ICG has also been used to nonspecifically detect cancers as a result of an enhanced permeability and retention (EPR) effect (13). The EPR effect for large particles (proteins, macromolecules, and liposomes) has been widely observed in solid tumors, mainly because of extensive angiogenesis, leaky vasculature, and impaired lymphatic drainage (14). However, this may not be ideal for the detection of ovarian cancer metastasis, owing to nonspecific signals (for example, inflammation) (15). The need for tumor-specific near-infrared agents remains.

A promising target for ovarian cancer is the gonadotropin-releasing hormone receptor (GnRHR). High-affinity GnRHR binding spots have been reported in 78% of ovarian cancers as well as in other hormone-related cancers (85% of endometrial cancers, 50% of breast cancers, and 86% of prostate cancers) (16, 17). Hence, GnRHR may be a good target for imaging purposes. As a ligand of GnRHR, GnRH peptide has already been conjugated to cytotoxic drugs or nanoparticles for targeted therapy and has shown strong antitumor activities.

In this study, we report the use of a molecular imaging probe using GnRH peptide, a specific and high-affinity GnRHR ligand, conjugated to the near-infrared fluorophore ICG. GnRHa-ICG showed specific binding capacities to GnRHR-positive cancer cells and effectively distinguished peritoneal metastases from adjacent normal tissue in ovarian cancer mouse models.

## Materials and Methods

### Analysis of the Cancer Genome Atlas (TCGA) Data

Transcriptome profiling data related to samples of ovarian cancer, breast cancer, endometrial cancer, and prostate cancer were downloaded from the TCGA data portal (2019). The GnRHR mRNA expression level is presented as FPKM-UQ (Upper Quartile normalized Fragments Per Kilobase to transcript per Million mapped reads).

### Cell Culture

A total of six cell lines were used for the experiments, including human ovarian cancer cell lines (A2780, CAOV-3, ES-2, and HeyA8), immortalized human normal ovarian surface epithelial (OSE) cells, and the human lung cancer cell line H1299. All cell lines were archived in our laboratory. Cells were cultured in RPMI-1640 medium containing 10% fetal bovine serum.

### Histology

Tissue microarrays comprising 56 high-grade serous ovarian cancer tissue samples were obtained from the tissue bank of the Obstetrics and Gynecology Hospital of Fudan University after Ethical Committee approval. The slides were stained with standard hematoxylin-eosin (HE) and immunohistochemistry (IHC) staining using a 1:100 dilution of GnRHR antibody (ab183079, Abcam). The GnRHR expression level was categorized as low, medium, or high.

### Western Blot

Protein lysates were obtained using cell lysis buffer supplemented with protease inhibitor. Proteins were separated by SDS-PAGE and transferred onto nitrocellulose filter membranes (Millipore). The membrane was incubated with the following primary antibodies: GnRHR antibody (Abcam, ab183079) and β-tubulin (Absin, abs830032). Proteins were visualized by chemiluminescence using the ImageQuant LAS4000 system (GE).

### Synthesis of GnRHa-ICG

The peptide sequence of GnRH antagonist Cetrorelix (D-2-Nal-D-4-Cl-Phe-D-3-Pal-Ser-Tyr-D-Cit-Leu-Arg-Pro-D-Ala-NH_2_) was adopted as the GnRHa peptide. GnRHa peptide (>95% purity) was synthesized using standard solid-phase methods (GL Biochem Ltd., Shanghai, China). Fluorescein isothiocyanate (FITC) was labeled on the N-terminus of the peptide by an Acp (amino caproic acid) linker. For the synthesis of GnRHa-ICG, GnRHa peptide (3 mg) and 20 μL of N, N-diisopropylethylamine were added into 0.5 mL of ultra-dry DMF and reacted for 10 min under nitrogen protection. ICG-NHS (1 mg) was added into the solution and continued reacted for 12 h at room temperature. The mixture was precipitated using 5 mL of ether. The obtained solid was dissolved with methanol and subjected to high performance liquid chromatography (HPLC) purification (Eluent: water/methanol) to obtain the green solid (2 mg). The final products of GnRHa-FITC and GnRHa-ICG were identified by analytical HPLC and MALDI mass spectroscopy.

### Fluorescence Microscopy

The cells were placed in an 8-well chamber slide (Ibidi) and grown to ~60% confluence. A2780, CAOV-3, ES-2, and HeyA8 cells were incubated with 100 μmol/L GnRHa-FITC, and A2780, OSE, and H1299 cells were incubated with 20 μmol/L GnRHa-ICG or ICG (Sigma-Aldrich) for 60 min at 37°C. After incubation, the cells were washed with PBS, fixed in 4% paraformaldehyde, labeled with WGA Alexa Fluor 594 conjugate or WGA Alexa Fluor 488 conjugate (Invitrogen), and mounted with DAPI. These samples were analyzed using TCS SP5 confocal microscopy (Leica). For quantitative analysis, the mean fluorescence intensity was calculated using ImageJ software (version 1.50 g).

### Flow Cytometry

A2780 and OSE cells were placed in 6-well plates, grown to ~70% confluence, and then incubated with GnRHa-FITC (20 μmol/L) or GnRHa-ICG/ICG (10 μmol/L) for 30 min at 37°C. For the blocking experiments, cells were pretreated with 100 μmol/L GnRHa peptide for 10 min and then incubated with 2 μmol/L GnRHa-ICG for 30 min at 37°C. Samples were measured on a CytoFLEX flow cytometer (Beckman Coulter) or a FACSAria flow cytometer (BD Biosciences). All samples were examined in triplicate. Data were analyzed using FlowJo software (version X 10.0.7).

### Cell Viability Assay

A2780 cells were seeded in 96-well plate and grown to ~40% confluence. Cells were incubated with GnRHa-ICG at different concentrations: 0, 1, 10, and 100 μmol/L. Each group had 4 sub-wells. After exposure for 48 h, cell viabilities were assessed by Cell Counting Kit-8 assay (Dojindo). The absorbance at 450 nm (reference wavelength: 630 nm) was measured with a microplate reader.

### Animal Model

The xenograft model for metastatic ovarian cancer was previously described (18). Female Balb/c nude mice (5–6 weeks old) were intraperitoneally injected with 1 × 10^7^ A2780 cells. Tumors were allowed to grow for 2–3 weeks. The Institutional Animal Care and Use Committee of Fudan University approved all protocols presented in these studies.

### Near-Infrared Fluorescence Imaging

For *ex vivo* GnRHa-ICG and ICG (0, 1, 10, and 20 μmol/L) imaging, fluorescence signals were taken using the IVIS Lumina K imaging system (PerkinElmer) and the clinically used Fluorescence Navigation system (the FloNavi, Optomedic Technique Inc., Guangdong, China). For intraperitoneal metastasis imaging, 0.72 μmol/kg of GnRHa-ICG or ICG was injected intraperitoneally in mice. Mice were sacrificed at the indicated times (*n* = 3 per group), and the abdominal cavities were exposed. Fluorescence images were obtained using the IVIS Lumina K imaging system (PerkinElmer) with a 780 nm excitation filter and an 845 nm emission filter. For *ex vivo* imaging, xenografts and organs were immediately dissected and analyzed after sacrifice.

Fluorescence signals were quantified as the average radiant efficiency ([p/s/cm^2^/sr]/[μW/cm^2^]) using the Living Image software. The fluorescence intensity was measured by drawing a region of interest (ROI) around the area. The tumor-to-background ratio (TBR) was calculated as the average fluorescence intensity of the tumor divided by that of the skeletal muscle or intestine.

### *In vivo* Toxicity Test

The toxicity of GnRHa-ICG was determined in Balb/c nude mice (*n* = 4). Two groups received intraperitoneal injections of 1.5 mg/kg GnRHa-ICG and were followed for 2 and 96 h. The control group received vehicle alone. Blood draws were done to assess alanine transaminase (ALT), aspartate transaminase (AST), blood urea nitrogen (BUN), creatinine (CREA), white blood cells (WBC), and red blood cells (RBC). Tissues of the heart, lung, liver, spleen and kidney were harvested for HE staining.

### Statistical Analysis

Student's *t*-tests were used to compare the intensities. Values of *P* < 0.05 were considered significant and reported as mean ± SD.

## Results

### GnRHR Is Overexpressed in Human Ovarian Cancer

**Figure 1:**
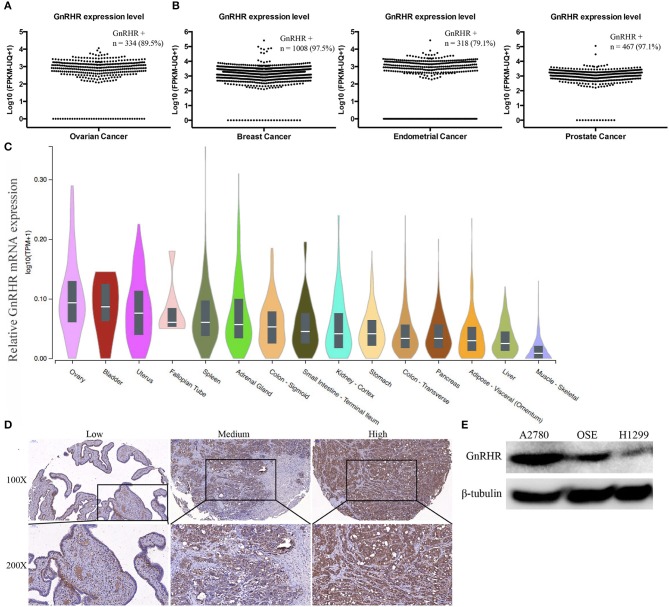
Gonadotropin-releasing hormone receptor is overexpressed in human ovarian cancer. Scatter plot of the GnRHR mRNA expression level in **(A)** serous ovarian cancer, **(B)** breast cancer, endometrial cancer, and prostate cancer from the TCGA dataset. **(C)** Relative GnRHR mRNA expression in human normal tissues. **(D)** Representative IHC staining for GnRHR in high-grade serous ovarian cancer. **(E)** Western blot showing GnRHR expression in different cell lines.

To evaluate the relevance of GnRHR as an imaging target in human ovarian cancer, we first analyzed 373 cases of serous ovarian cancer from TCGA datasets. GnRHR mRNA expression was found in 89.5% of these samples (Figure 1A). In addition, 97.5% of breast cancers, 79.1% of endometrial cancers, and 97.1% of prostate cancers in the TCGA datasets also expressed GnRHR (Figure 1B), suggesting that GnRHR targeting may be effective in other hormone-related tumors. In addition, we analyzed GnRHR expression in various normal tissues from the Genotype-Tissue Expression (GTEx) Portal database. Higher expression of GnRHR mRNA was observed in ovaries than in other tissues in the abdominal and pelvic cavities (Figure 1C). GnRHR expression was confirmed by IHC analysis of 56 high-grade serous ovarian carcinomas from our tissue bank. Moderate (10 cases, 18%) and high (43 cases, 77%) expression of GnRHR was observed in 95% of these tumors (Figure 1D), consistent with previous studies (17, 19). Collectively, these data suggested that GnRHR could be a potential imaging target for the detection of ovarian cancer.

GnRHR expression in cancer cell lines was evaluated by western blot. The ovarian cancer cell line A2780 and immortalized normal OSE cells showed high expression levels; the lung cancer cell line H1299 showed low expression levels (Figure 1E). Therefore, we selected A2780 and OSE as the positive controls and H1299 as the negative control for further studies.

### Synthesis and Characterization of GnRHa-ICG

**Figure 2:**
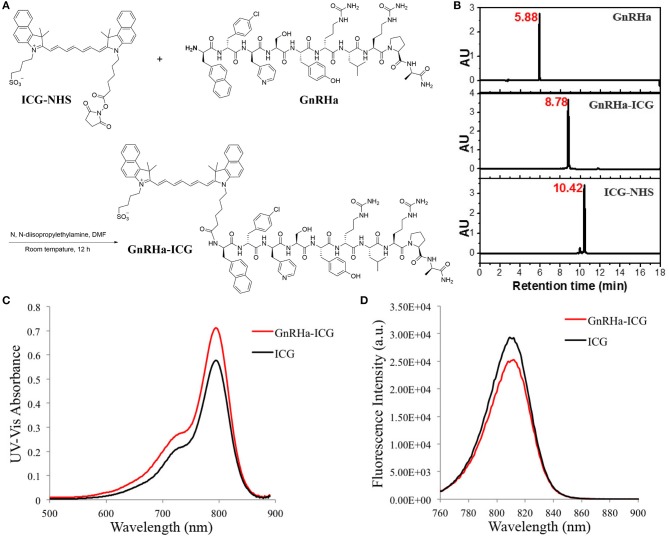
ICG conjugation of the GnRHa peptide. **(A)** Synthetic route of GnRHa-ICG. **(B)** HPLC analysis of GnRHa-ICG. **(C,D)** UV-Vis absorbance and fluorescence spectra of GnRHa-ICG.

ICG was conjugated to the GnRHa peptide (D-2-Nal-D-4-Cl-Phe-D-3-Pal-Ser-Tyr-D-Cit-Leu-Arg-Pro-D-Ala-NH_2_). We chose the peptide sequence of Cetrorelix, a widely used GnRHR antagonist, which has several amino acids modified from the natural hypothalamic hormone (20). A schematic diagram of the molecular structure is shown in Figure 2A. The final product was identified by analytical HPLC (Figure 2B). The molecular weight of GnRHa-ICG was 2082 Da. The conjugated near-infrared fluorophore had an excitation wavelength of 795 nm and emitted light at 810 nm (Figures 2C,D). *Ex vivo* fluorescence imaging of GnRHa-ICG and ICG was obtained using the IVIS Lumina K imaging system (Figure S1A) and clinically used Fluorescence Navigation System (Figure S1B).

### The Enhanced Cell Binding Capacity of GnRHa-ICG

**Figure 3:**
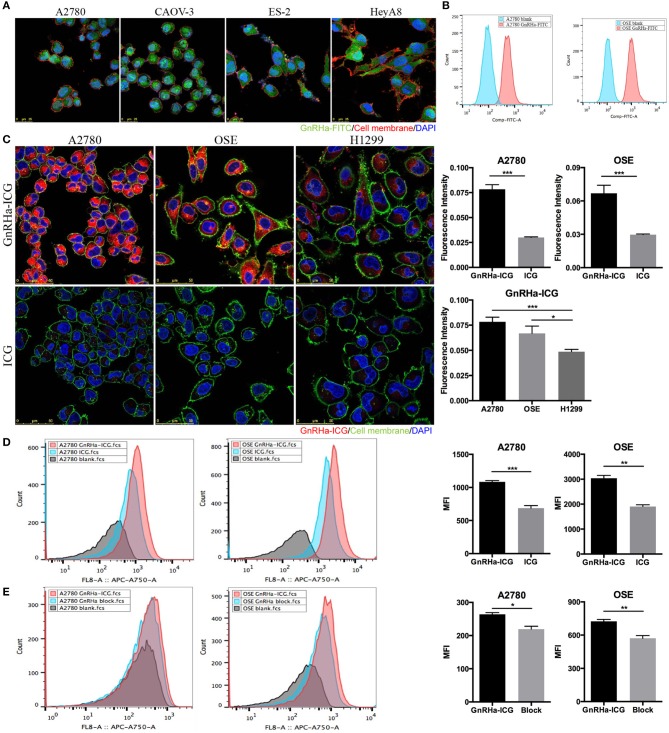
Cell binding study of GnRHa-ICG. **(A)** Representative fluorescence microscopy images of ovarian cancer cells after incubation with GnRHa-FITC. **(B)** Flow cytometry analysis of A2780 and OSE cells after incubation with GnRHa-FITC. **(C)** Comparison between GnRHa-ICG and ICG in A2780, OSE, and H1299 cells. **(D)** Flow cytometry analysis of A2780 and OSE cells after incubation with GnRHa-ICG and ICG. **(E)** Flow cytometry analysis of A2780 and OSE cells after the GnRHa block (*P < 0.01; **P < 0.001; ***P < 0.0001).

To test whether GnRHa peptide was taken up by ovarian cancer cells, we incubated GnRHa-FITC with different cell lines. Fluorescence signals were detected in different ovarian cancer cell lines (Figure 3A). Flow cytometry analysis confirmed the accumulation of GnRHa-FITC in A2780 and OSE cells (Figure 3B).

Next, we incubated GnRHa-ICG with cancer cell lines with different GnRHR expression levels. A significant difference in fluorescence intensity was detected between the GnRHR-positive cell lines (A2780 and OSE) and the GnRHR-negative cell line (H1299) (Figure 3C). The fluorescence intensity in the A2780 and OSE cells was 1.56- and 1.34-fold higher, respectively, than that in the H1299 cells. To control for nonspecific binding, incubation with ICG alone was performed. A significant difference in the fluorescence signal was observed between ICG (20 μM) and GnRHa-ICG (20 μM) (Figure 3C). The fluorescence signal was 2.61 and 2.25 times lower than that with the incubation of GnRHa-ICG in A2780 and OSE cells, respectively. We further confirmed the accumulation of GnRHa-ICG in GnRHR-positive cells by flow cytometry analysis (Figure 3D). In addition, competition of GnRHa-ICG with unlabeled GnRHa resulted in a partial reduction in fluorescence intensity (Figure 3E). To address concerns of possible side effects caused by GnRHa-ICG, we assessed the tumorigenic potential of GnRHa-ICG exposure by assessing the cell viability of GnRHR positive A2780 cells. Our results indicated that a 48-h exposure does not increase cell viability (Figure S2). Taken together, these results demonstrated the GnRHR-specific binding capacity of GnRHa-ICG.

### GnRHa-ICG Specifically Recognized Peritoneal Metastases of Ovarian Cancer

**Figure 4:**
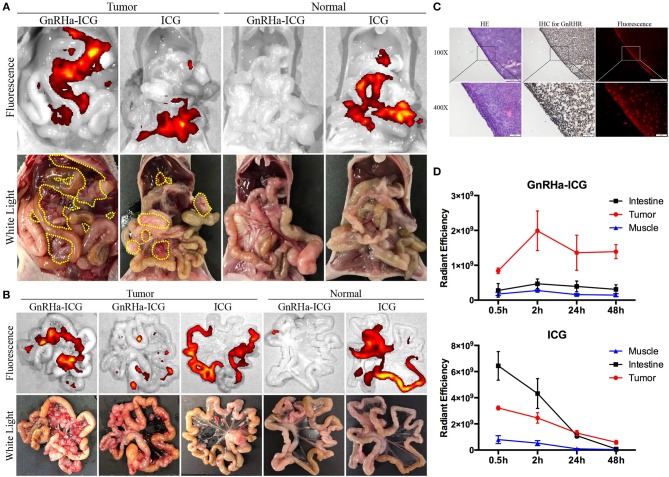
GnRHa-ICG localizes to GnRHR expression in peritoneal ovarian cancer. Representative images of the **(A)** peritoneal cavity and **(B)** mesentery 2 h after the injection of GnRHa-ICG and ICG. Yellow dotted lines indicate the tumor location. **(C)** Histopathological analysis of a tumor slice showing the colocalization of tumor cells, GnRHR expression, and GnRHa-ICG fluorescence. **(D)** Comparison of fluorescence intensities between the tumor, muscle, and intestine.

Common metastasis sites of ovarian cancer include the omentum, mesentery, and intestine. To evaluate its specificity for *in vivo* cancer imaging, we injected GnRHa-ICG and ICG intraperitoneally into a mice model of metastatic ovarian cancer and control mice. Mice were sacrificed 2 h after injection, and fluorescence images were obtained and NIRF signals analyzed using the IVIS Lumina K imaging system (Figure 4A). Separate fluorescence intensities of the tumor and background (muscle and intestine) per dose group are shown in Figure S3. A dose of 1.5 mg/kg was considered for the following experiments because of the higher TBR. In tumor-bearing mice, GnRHa-ICG specifically localized to peritoneal tumors, while accumulation of ICG was observed not only at the tumor site but also in the intestine. Control mice injected with GnRHa-ICG did not show fluorescence signals, which indicated a low background in normal tissues. In contrast, the intestine exhibited high fluorescence signals in normal mice injected with ICG. The corresponding *ex vivo* imaging of the intestine with mesenteric metastasis is shown in Figure 4B. Tiny tumor nodules (~1 mm) on the mesentery could also be visualized while the intestine exhibited few fluorescence signals after GnRHa-ICG administration. The intraperitoneal lesions were also confirmed by pathology and exhibited GnRHR overexpression and the accumulation of GnRHa-ICG (Figure 4C).

Metastases of ovarian cancer are widely spread in the pelvic and abdominal cavities, and both the intestine and muscle were considered as the background. To further evaluate the capacity of GnRHa-ICG for distinguishing peritoneal metastases from background tissues, the fluorescence intensities of the tumor, intestine, and muscle were collected at 0.5, 2, 24, and 48 h (Figure 4D). GnRHa-ICG exhibited few fluorescence signals in the intestine and muscle tissues at any time point. ICG exhibited higher fluorescence signals in the intestine tissues than in tumors before 24 h and similar fluorescence signals in both tissues after 24 h. The TBR is shown in Figure S4. The TBR of GnRHa-ICG was stable between 2 and 48 h. Although the TBR of ICG increased after 24 h because of the EPR effect and fast clearance, the fluorescence intensities of the tumor decreased quickly and might have influenced the detection of tumor signals. Furthermore, according to a previous study, the clinical use of ICG based on the EPR effect was not viable owing to a high false-positive rate, especially in lymph node and inflammatory tissues (15). In the present study, *ex vivo* imaging of the brain did not show fluorescence signals compared to the tumor and ovarian tissues, which indicated limited binding of GnRHa-ICG to the hypothalamic GnRHR receptor (Figures S5A,C). In addition, fewer fluorescence signals were detected in the benign retroperitoneal lymph nodes than in the tumor (Figures S5B–C).

These data suggested that GnRHa-ICG could specifically recognize metastatic lesions from peritoneal normal tissues and intestine tissues, and the modification of GnRHa improved the capacity of ICG for tumor imaging.

### Dynamics and Biodistribution of GnRHa-ICG

**Figure 5:**
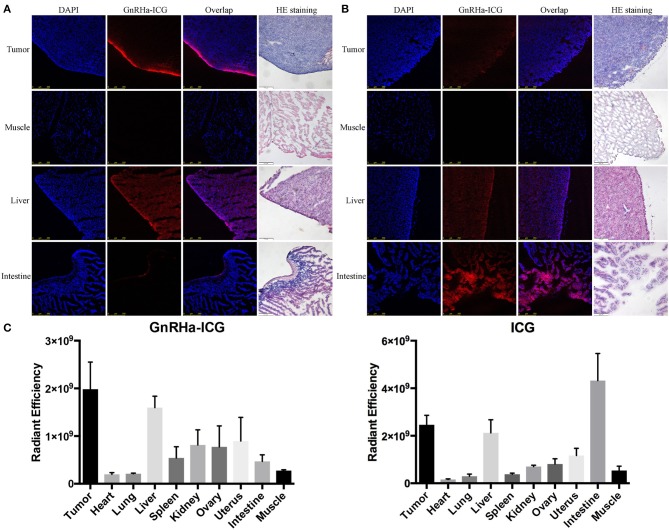
Comparison of biodistribution between GnRHa-ICG and ICG. Confocal microscopy of frozen sections of tumors and other organs in mice injected with **(A)** GnRHa-ICG and **(B)** ICG. **(C)** Biodistribution of GnRHa-ICG and ICG 2 h after injection.

To evaluate the biodistribution of probes, the fluorescence signals of *ex vivo* tumor and organs were collected at 2 h post administration. Confocal imaging of A2780 xenograft confirmed the high accumulation of GnRHa-ICG, while ICG also demonstrated a weak signal in the tumor, mainly because of leaky vasculature and impaired lymphatic drainage in the tumor tissues (EPR effect) (Figures 5A,B). Additionally, ICG showed strong fluorescence signals in the liver and intestine. These results were consistent with the *in vivo* fluorescence signal. As shown in Figure 5C, a biodistribution assay of ICG also showed high fluorescence in the liver and intestine, indicating liver-intestine clearance, while GnRHa-ICG showed low fluorescence in the intestine.

**Figure 6:**
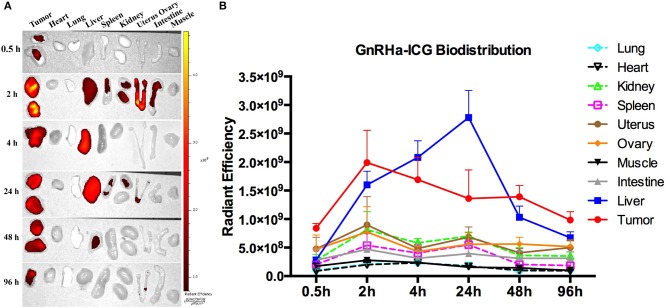
Dynamics and biodistribution of GnRHa-ICG. **(A)** Representative *ex vivo* fluorescence images of A2780 xenografts and mouse organs. **(B)** Biodistribution of GnRHa-ICG.

The metabolism of GnRHa-ICG in A2780 tumor-bearing mice was further evaluated. Mice were injected intraperitoneally with GnRHa-ICG and monitored for 96 h. Representative *ex vivo* NIRF images of different time points are presented (Figure 6A). The mean fluorescence intensities of the xenografts and organs of different time points are shown (Figure 6B). GnRHa-ICG was cleared mainly through the liver pathway and reached the highest fluorescence density in the liver at 24 h post administration. The fluorescence signal of the A2780 xenografts peaked in intensity at 2 h. The tumor fluorescence intensities were maintained for up to 48 h and decreased slightly after that. The plateau period (from 2 to 48 h) of GnRHa-ICG specific accumulation may be feasible for clinical applications in cytoreductive surgery. Although normal uterus and ovary tissues also showed a slight increase in fluorescence signals, indicating GnRHR-specific binding of the probe, the intensities of malignant lesions were high enough to differentiate them from those of normal reproductive tissues. Few fluorescence signals were detected in the intestine, kidney, and other normal tissues.

### Toxicity of GnRHa-ICG

**Figure 7:**
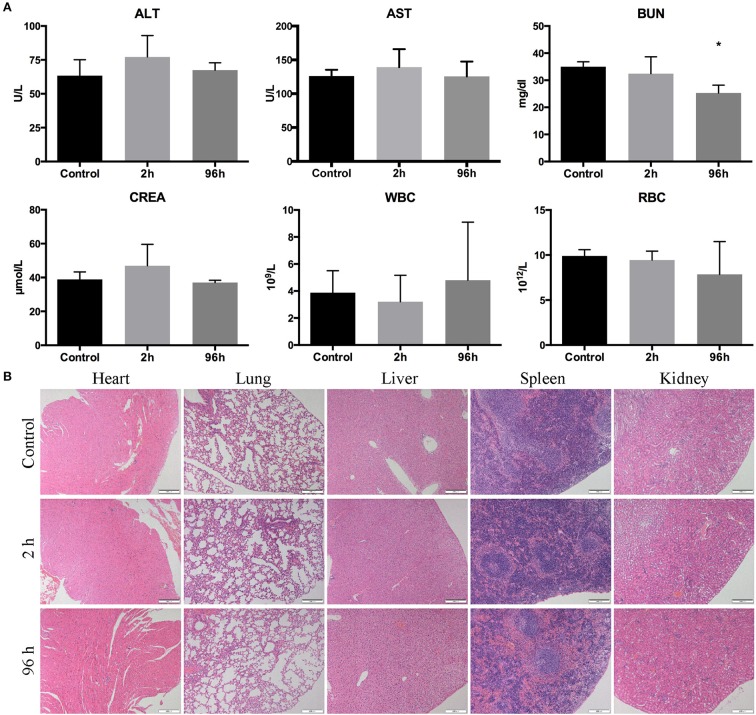
Toxicity of GnRHa-ICG in Balb/c nude mice. **(A)** Assessment of the liver panel, kidney panel, and blood cells. **(B)** HE staining of the heart, lung, liver, spleen, and kidney (*P < 0.05).

Toxicity of GnRHa-ICG was determined in Balb/c nude mice (*n* = 4) by measurement of blood panels and hematology. There was no significant difference observed in ALT, AST, CREA, WBC, and RBC between the control and test groups. A slight decrease in BUN was observed 96 h after GnRHa-ICG injection, which may be attributable to dietary factors (Figure 7A). Histological observation of different main organs did not present any obvious damage (Figure 7B).

## Discussion

Herein, we report a novel targeted imaging probe, near-infrared fluorophore GnRHa-ICG, and its successful use to target peritoneal metastases of ovarian cancer in mice models, suggesting a strong potential for the application of fluorescence-guided surgery.

Over the past decade, several studies have demonstrated the potential use of fluorescence-guided surgery in ovarian cancer. Although studies have shown the capacity to detect ovarian cancer in real time, existing probes still have some deficiencies, including poor specificity, insufficient penetration depth, and side effects. Van Dam reported the first human trial of folate receptor α-targeted imaging in ovarian cancer (9). Although this study showed the real-time intraoperative detection of tumor deposits, the imaging agent FITC has a limited penetration depth due to its non-NIR fluorescence spectrum. Vahrmeijer replaced FITC with the NIRF dye Cy7 (OTL38), which improved the penetration depth, but the agent showed nonspecific binding in noncancerous lymph nodes (express folate receptor β) (10). False-positive lymph nodes were also observed in a phase II trial of OTL38 in ovarian cancer (21). Sundaram reported a prolactin receptor-specific probe using placental lactogen (hPL) conjugated to MRI and NIRF imaging agents and showed improved specificity over currently used contrast agents (11). However, continuous exposure (16 days) to hPL conjugates caused increased activity in ovarian cancer cells. Thus, the demand for ovarian cancer-specific near-infrared probes remains.

A significant number of studies on GnRHR-based therapy have substantiated its applicability, specificity, and safety in cancer targeting (16). In this study, we introduced GnRHR as the target for the molecular imaging of ovarian cancer. It is well established that GnRHR is overexpressed in ovarian cancer and other hormone-related cancers, even in some hormone-unrelated cancers (pancreatic cancer, lung cancer, melanoma, and glioblastoma) (17). The expression of GnRHR in ovarian cancer tissue is higher than that in normal ovarian tissue (22). In addition, GnRHR expression is also detected in lymph node metastasis (23). Consistent with previous studies, our own analysis confirmed that the majority (95%) of high-grade serous ovarian cancers express moderate to high levels of GnRHR. Having demonstrated the specific binding capacity of GnRHa-ICG in ovarian cancer, we speculate that our GnRHR imaging concept might also be effective in other tumors with upregulated GnRHR expression.

Currently, a few viable targets, including the folate receptor and prolactin receptor, have emerged for optical imaging in ovarian cancer. To compare the specificity of these targets, we analyzed the expression of different receptors in normal tissues within the abdominal and pelvic cavities, which may be involved in debulking surgery (GTEx Portal database, data not shown). Higher expression of GnRHR was observed in reproductive tissues than in other tissues. However, higher expression of the folate receptor and prolactin receptor was observed in the gastrointestinal tract. These results suggested that GnRHR might have better specificity than the folate or prolactin receptors. The Lymphadenectomy in Ovarian Neoplasms (LION) trial reported that the removal of clinically negative lymph nodes was not associated with longer survival and may cause excess morbidity and mortality (24). Thus, non-specific binding of noncancerous lymph nodes should be avoided. Compared to the previously reported folate receptor α-targeted probe OTL38 (10, 21) or the clinically used non-specific imaging agent ICG (15), non-specific binding to benign retroperitoneal lymph nodes was not observed using GnRHR-ICG in our mice model of metastatic ovarian cancer.

As the binding ligands of GnRHR, GnRH analogs (both agonists and antagonists) have been conjugated to various cytotoxic drugs and shown a high affinity for cancer cells. Few studies have employed GnRH analogs to achieve targeted delivery of optical imaging agents (25–27). Existing GnRHR-targeted agents use GnRH agonists as the binding moiety and have not been validated in peritoneal metastasis models. Although many studies have demonstrated the antitumor activity of GnRH agonists, contrasting results also exist. Schally's group reported that a [D-Trp^6^] GnRH agonist stimulated the proliferation of ovarian cancer cells at a low dose (28). Unlike agonists, antagonists competitively bind to GnRHR without activating the downstream signaling cascade. Several GnRH antagonists are already commercially available and have various clinical applications. Thus, we adopted the peptide sequence of Cetrorelix, a third-generation antagonist, as the targeting sequence of the imaging agent. Cetrorelix has been reported to inhibit the growth of ovarian, endometrial, breast, and prostate cancer, suggesting a potential therapeutic effect as the targeting moiety (17). In the current study, no obvious tumorigenic potential caused by GnRHa-ICG exposure was observed in the *ex vivo* experiments. In terms of the concern of potential binding with the hypothalamic GnRHR, fluorescence imaging of brain indicated limited binding of GnRHa-ICG in the hypothalamus, which may be due to the peritoneal administration of the drugs performed in this study. Further animal studies are warranted to assess the tumorigenic or endocrine potential of GnRHa-ICG.

NIRF imaging is an emerging biomedical imaging modality for fluorescence-guided surgery because of its significant penetration depth, light absorption, real-time capabilities, and absence of ionizing radiation. The first FDA-approved NIRF dye, ICG, has been in clinical use for more than half a century and has proven to be safe and feasible. GnRHa-ICG had the same excitation and emission wavelength as ICG, which made its clinical application possible. Owing to the EPR effect, ICG has been used for the intraoperative identification of cancers, including ovarian, pancreatic, and colorectal cancer (8). However, the ICG detection of ovarian cancer and metastasis is not satisfactory because of nonspecific binding. Tummers reported that despite the successful detection of all metastatic lesions, 13 nonmalignant lesions also exhibited fluorescence, resulting in a high false-positive rate of 62% (15). A recent pilot study revealed that ICG could detect peritoneal metastasis but was unable to distinguish between benign and malignant nodules after neoadjuvant chemotherapy (29). Previous studies have highlighted the need for the development of more specific probes to detect metastatic ovarian cancer.

In this study, we developed a tumor-specific probe, GnRHa-ICG, by conjugating ICG with a GnRH antagonist peptide. Both ICG and the GnRH antagonist (Cetrorelix) were already proven to be safe in clinical use. GnRHa-ICG showed specific binding to peritoneal metastases in ovarian cancer mouse models, whereas ICG was mainly localized to the liver and intestine, consistent with its liver-intestine clearance. ICG also exhibited a weak signal in tumors due to the EPR effect, but the intensity decreased quickly within 48 h. In contrast, the plateau period of GnRHa-ICG accumulation in metastases lasted for nearly 2 days and allowed for flexibility in terms of surgery time, which may be practical for clinical applications. Both ICG and Cetrorelix were cleared through the liver-intestine pathway. The conjugation of GnRHa (the peptide sequence of Cetrorelix) to ICG may have affected the pharmacokinetic and biodistribution properties of the ICG. Liver metabolism of GnRHa-ICG was significantly decreased compare to that of ICG. A slight increase in intestinal signals within the subsequent 96 h indicated that the drug might be cleared through biliary excretion. Whether there are other metabolic pathways remains to be investigated. The toxicity of GnRHa-ICG was assessed, and no obvious hepatotoxicity was observed in the current study.

Previous preclinical studies have developed several ICG-based targeted imaging agents. BLZ-100 is a tumor-targeted imaging agent composed of ICG and the modified CTX peptide, which targets Annexin A2 on cancer cells (30). BLZ-100 was successfully validated in canine tumor models and is being evaluated in phase I clinical trials. Furthermore, Ogawa conjugated ICG to three FDA-approved monoclonal antibodies (daclizumab, trastuzumab, and panitumumab) (31), and a prostate-specific membrane antigen (PSMA)-specific imaging probe was synthesized by linking ICG to the anti-PSMA antibody J591 (32). In contrast to previous studies, we utilized peritoneal rather than systemic administration of the imaging agent. Since ovarian cancer has a peritoneal dissemination pattern, imaging agents can bind to malignant lesions more directly through peritoneal injection. Several studies have testified the viability of the peritoneal administration of imaging agents for detecting peritoneal metastasis (11, 33, 34). Nonetheless, systemic administration of the probe will be attempted to detect distant metastasis or deep lesions in further studies.

Several limitations exist in this study. Further assessment of the tumorigenic or endocrine potential of GnRHa-ICG is needed in molecular levels. The metabolic pathways of GnRHa-ICG remain unclear. The specificity and sensitivity of GnRHa-ICG imaging for ovarian cancer lesions should be investigated in a pilot study.

In summary, the developed GnRHR-targeting imaging agent binds selectively to ovarian cancer. The GnRHa-ICG probe has the potential to aid surgeons in staging and debulking surgery via intraoperative tumor-specific fluorescence imaging. Our GnRHR imaging concept may also be effective in other hormone-related tumors with upregulated GnRHR expression.

## Data Availability Statement

The datasets used in this study are available at: TCGA: https://portal.gdc.cancer.gov.

Selection criteria: Primary site: Ovary/Breast/Prostate gland/Corpus uteri; Workflow Type: HTSeq-FPKM-UQ; Data Category: Transcriptome Profiling; Experimental Strategy: RNA-Seq.

GTEx Portal: https://gtexportal.org/home/.

Gene symbol: GnRHR ENSG00000109163; FSHR ENSG00000170820.

## Ethics Statement

The Institutional Animal Care and Use Committee of Fudan University approved all protocols presented in these studies.

## Author Contributions

QL: experimental studies, data analysis, and writing—original draft. XZho: experimental studies and data analysis. WF, TP, XL, FL, and YK: experimental studies and methodology. XZha and CX: study design and writing—review and editing.

### Conflict of Interest

The authors declare that the research was conducted in the absence of any commercial or financial relationships that could be construed as a potential conflict of interest.

## Supplementary Material

Figure S1 *Ex vivo* fluorescence imaging of GnRHa-ICG and ICG (0, 1, 10, and 20 μmol/L) using the IVIS Lumina K imaging system and clinically used Fluorescence Navigation system. **(A)** Fluorescence images of the IVIS Lumina K imaging system. **(B)** Fluorescence images of the Fluorescence Navigation system.

Figure S2 Cell viability of A2780 cells treated with GnRHa-ICG. Samples include no exposure to GnRHa-ICG (control) and a 48-h exposure to different concentrations of GnRHa-ICG.

Figure S3 Dose-escalation cohort of GnRHa-ICG. **(A)** Mean fluorescence intensities of the tumor and background tissues. **(B)** Tumor-to-background ratio per dose group.

Figure S4 Tumor-to-background ratio of GnRHa-ICG and ICG at different time points. **(A)** Tumor-to-intestine ratio. **(B)** Tumor-to-muscle ratio.

Figure S5 Limited binding of GnRHa-ICG in the brain and lymph node tissues. **(A)** *Ex vivo* imaging of the hypothalamus (yellow arrow). **(B)** *Ex vivo* imaging of lymph node tissues (yellow arrow). **(C)** Histopathological analysis of the hypothalamus and lymph node tissues. Liver tissue was used as a positive control.
