# Supplementary figures and images for: Gonadotropin-Releasing Hormone Receptor-Targeted Near-Infrared Fluorescence Probe for Specific Recognition and Localization of Peritoneal Metastases of Ovarian Cancer
Source: Front Oncol. 2020 Feb 28;10:266. doi: 10.3389/fonc.2020.00266 (PMC7059204; doi:10.3389/fonc.2020.00266)

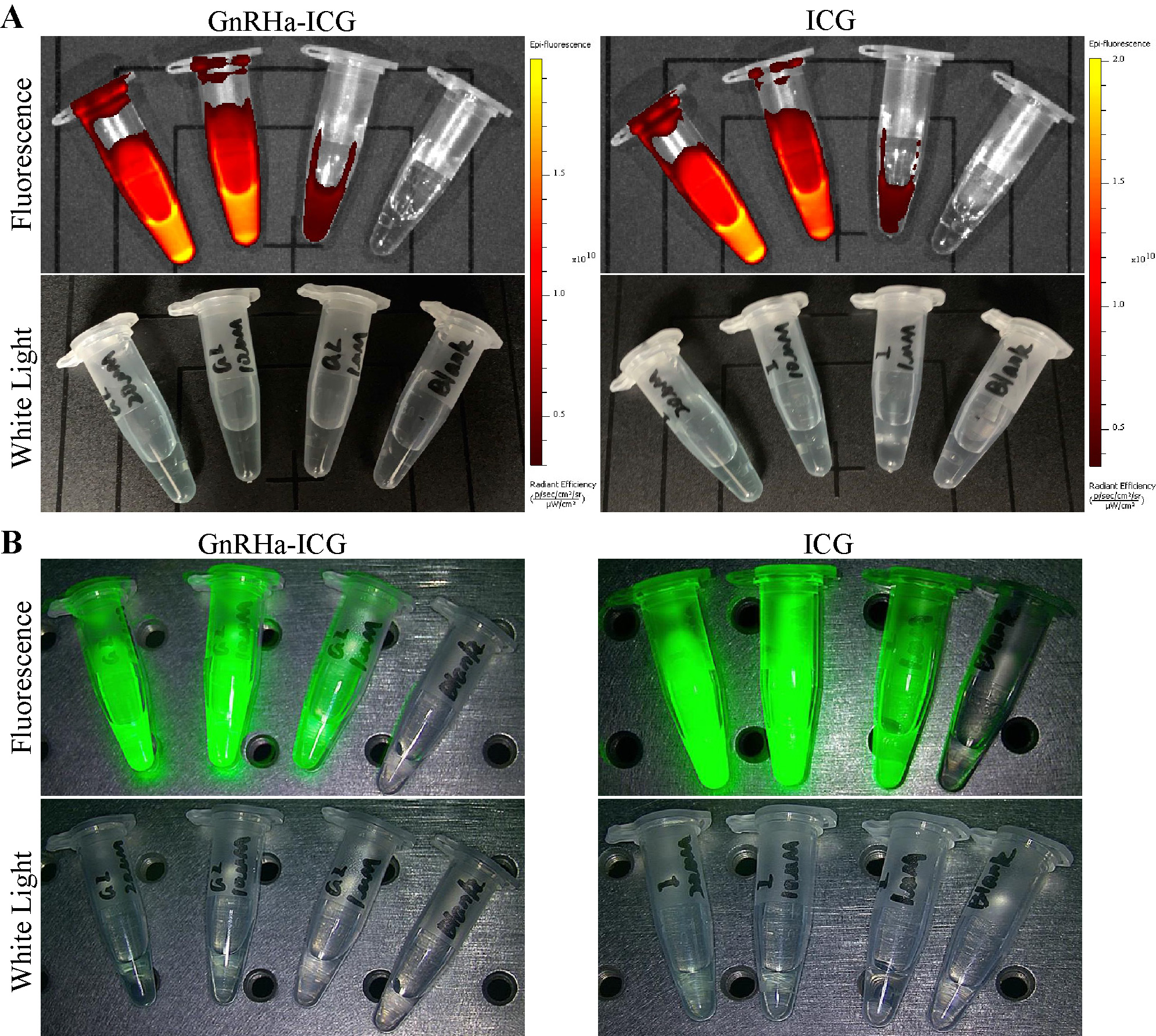

Supplement: Figure S1 — Ex vivo fluorescence imaging of GnRHa-ICG and ICG (0, 1, 10, and 20 μmol/L) using the IVIS Lumina K imaging system and clinically used Fluorescence Navigation system. (A) Fluorescence images of the IVIS Lumina K imaging system. (B) Fluorescence images of the Fluorescence Navigation system. [file Image_1.JPEG]

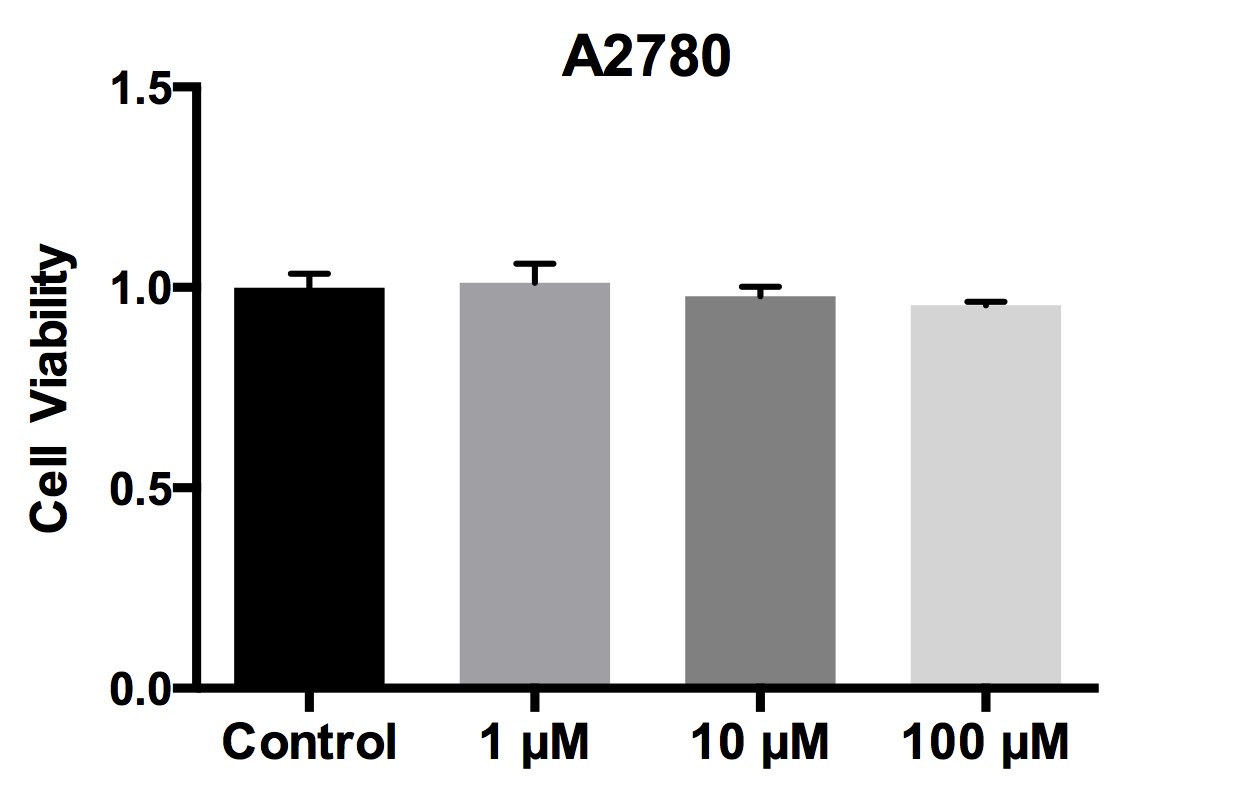

Supplement: Figure S2 — Cell viability of A2780 cells treated with GnRHa-ICG. Samples include no exposure to GnRHa-ICG (control) and a 48-h exposure to different concentrations of GnRHa-ICG. [file Image_2.JPEG]

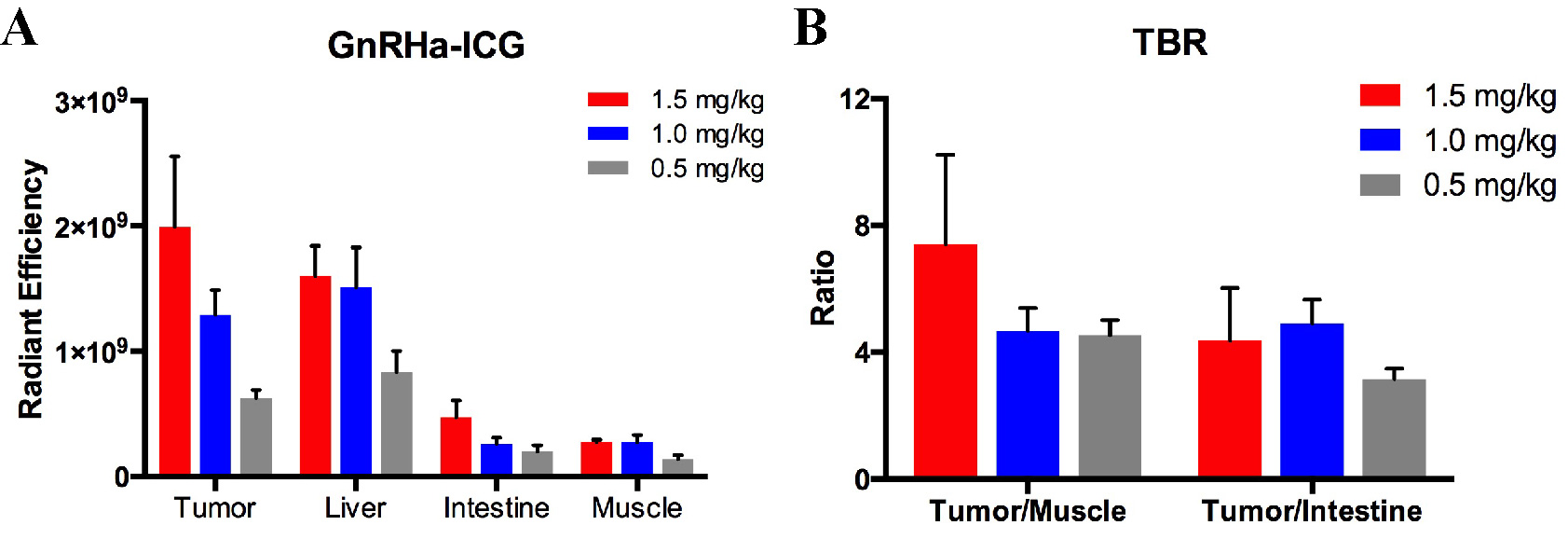

Supplement: Figure S3 — Dose-escalation cohort of GnRHa-ICG. (A) Mean fluorescence intensities of the tumor and background tissues. (B) Tumor-to-background ratio per dose group. [file Image_3.JPEG]

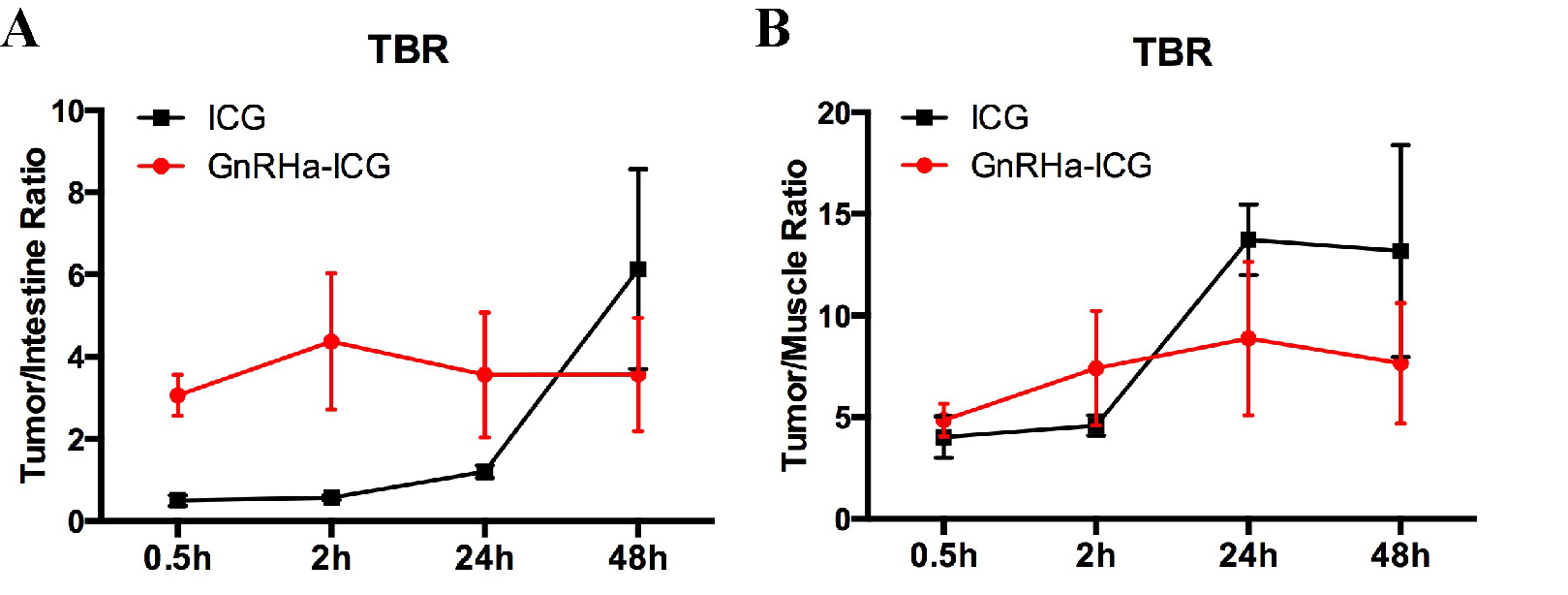

Supplement: Figure S4 — Tumor-to-background ratio of GnRHa-ICG and ICG at different time points. (A) Tumor-to-intestine ratio. (B) Tumor-to-muscle ratio. [file Image_4.JPEG]

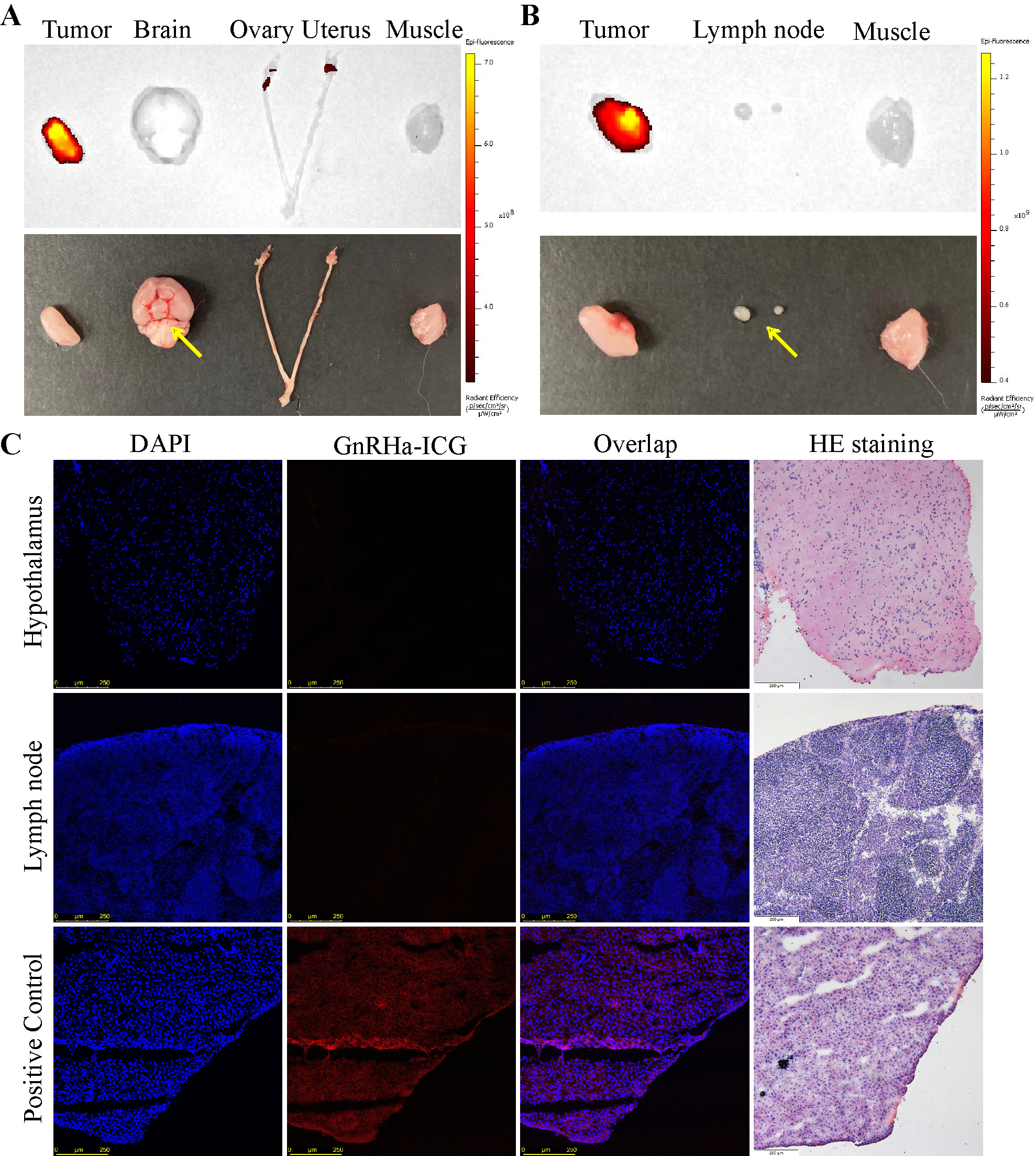

Supplement: Figure S5 — Limited binding of GnRHa-ICG in the brain and lymph node tissues. (A) Ex vivo imaging of the hypothalamus (yellow arrow). (B) Ex vivo imaging of lymph node tissues (yellow arrow). (C) Histopathological analysis of the hypothalamus and lymph node tissues. Liver tissue was used as a positive control. [file Image_5.JPEG]
